# T Cell Extracellular Traps: Tipping the Balance Between Skin Health and Disease

**DOI:** 10.3389/fimmu.2022.900634

**Published:** 2022-06-20

**Authors:** Kelsey Ouyang, Nicole Oparaugo, Amanda M. Nelson, George W. Agak

**Affiliations:** ^1^ Cleveland Clinic Lerner College of Medicine of Case Western Reserve University, Cleveland, OH, United States; ^2^ Division of Dermatology, Department of Medicine, University of California, Los Angeles (UCLA), Los Angeles, CA, United States; ^3^ David Geffen School of Medicine at University of California, Los Angeles (UCLA), Los Angeles, CA, United States; ^4^ Department of Dermatology, Penn State University College of Medicine, Hershey, PA, United States

**Keywords:** T cell extracellular traps, acne, *Cutibacterium acnes* (*C acnes*), T_H_17 cell, skin

## Abstract

The role of extracellular traps (ETs) in the innate immune response against pathogens is well established. ETs were first identified in neutrophils and have since been identified in several other immune cells. Although the mechanistic details are not yet fully understood, recent reports have described antigen-specific T cells producing T cell extracellular traps (TETs). Depending on their location within the cutaneous environment, TETs may be beneficial to the host by their ability to limit the spread of pathogens and provide protection against damage to body tissues, and promote early wound healing and degradation of inflammatory mediators, leading to the resolution of inflammatory responses within the skin. However, ETs have also been associated with worse disease outcomes. Here, we consider host-microbe ET interactions by highlighting how cutaneous T cell-derived ETs aid in orchestrating host immune responses against *Cutibacterium acnes (C. acnes)*, a commensal skin bacterium that contributes to skin health, but is also associated with acne vulgaris and surgical infections following joint-replacement procedures. Insights on the role of the skin microbes in regulating T cell ET formation have broad implications not only in novel probiotic design for acne treatment, but also in the treatment for other chronic inflammatory skin disorders and autoimmune diseases.

## Introduction

The skin is the largest organ in the body, and it provides an effective physical and immune barrier between the internal and external environment. As the skin is under constant assault from physical, chemical and biological threats, disturbance of this barrier can manifest as inflammatory skin diseases, such as atopic dermatitis and acne vulgaris. Contributing to the healthy skin barrier is a collection of diverse skin microbiota, which blanket the skin’s surface and populate the hair follicles, helping to promote skin homeostasis, immune defense and education of host immune cells (1, 2). Hair follicles penetrate deep into the dermis and contain a diverse population of commensal bacteria in comparison to the skin surface (1, 2). Although the hair follicle is an immune-privileged site, it may serve as an important site for interactions between bacteria and host T cells, particularly in acne, wherein the epithelium of the follicles and sebaceous glands are breached. In the healthy state, little is known about the potential for bi-directional movement of immune cells, including T cells from the dermis through the intact follicular epithelium to contact luminal bacteria. This interaction between immune cells and the diverse skin bacteria may contribute to both skin homeostasis and/or disease.

Human skin contains abundant populations of memory αβT cells, antigen presenting cells (APCs), natural killer cells, γδ T cells, and innate lymphoid cells (3). Effector and memory T cells within the skin coordinate immune responses against microbes. However, inappropriate T cell activation against innocuous or autoantigens can lead to chronic skin inflammatory disorders. In this review, we focus on the immune responses mediated by skin-resident T_H_17 cells that develop in response to *Cutibacterium acnes* (*C. acnes*), a bacterium that has been associated with acne vulgaris. We discuss the antimicrobial capacity of these cells in acne resolution and/or inflammation (4). The etiology and pathogenesis of acne vulgaris is briefly discussed, followed by the CD4^+^ T cell responses against *C. acnes* and their ability to form extracellular traps (ETs) in response to microbial threats. On this basis, mechanisms of T cell extracellular trap (TET) formation are proposed and the dual nature of ET formation in acne and select skin diseases is detailed, with a focused discussion on the specific factors that may contribute to host protection versus exacerbation of disease. We highlight new perspectives into how healthy skin commensals are critical to the instruction of our immune system and how T cell-derived–antimicrobial molecules may provide new therapeutic strategies in our overall defense against pathogens. The elucidation of subcellular events leading to TET formation may generate novel therapeutic strategies that target both the innate and adaptive immune system in order to ameliorate skin diseases.

## Etiology and Pathogenesis of Acne

Acne vulgaris is a chronic inflammatory disorder of the pilosebaceous unit (PSU) that most commonly occurs during adolescence but may linger into adulthood (5–8). In most cases, this skin disease is characterized with an array of lesions, which consist of comedones, papules, pustules, and nodules that are concentrated on the face, back, or chest. While the course of acne progression may be limited in most patients, the aftereffects can be life-long, with physical scars and psychological impairment, especially in adolescents (9). The pathogenesis of acne involves several factors, and at least four factors have been identified. These key factors include: follicular epidermal hyperproliferation, sebum production, C. acnes (in italics), and inflammation/immune response. Each of these processes are interrelated and can be influenced by both hormones and the immune response (9–15).

Current evidence supports the notion that acne lesions begin with the formation of the microcomedo with subsequent development into clinically detectable lesions and scarring, in the most severe cases. Follicular epidermal hyperproliferation contributes to microcomedo formation. During lesion development, the microcomedo expands with a densely packed layer of keratin, and there is increased sebum production that supports bacteria growth within the PSU. Eventually this ballooning effect causes the walls of the follicles to rupture leading to extrusion of keratin, sebum, and bacteria into the surrounding dermis, triggering a rapid inflammatory response. The predominant cell type present within 24 hours of rupture is the lymphocyte. CD4^+^ T lymphocytes are present around the ruptured PSU, while CD8^+^ T cells are found within the perivascular region. One to two days after the comedo is ruptured, neutrophils become the main cell type surrounding the ruptured microcomedo (9–15), further amplifying skin inflammation.

It is generally accepted that acne does not occur without sebum. Sebaceous gland activity increases during puberty in response to androgens (16). The anaerobic and lipophilic microenvironment of the PSU favors the growth of *C. acnes* over other skin microbes. Sebum, the lipid-rich fluid of sebaceous glands, serves as the primary nutrient source for *C. acnes.* Breakdown of sebum triglycerides into free fatty acids by *C. acnes* contributes to the inflammatory response (17, 18).

*C. acnes* also contributes to skin inflammation through activation of the immune response. *C. acnes*-induced secretion of proinflammatory cytokines from monocytes involves Toll-like receptor 2 (TLR2) (19), which is expressed on macrophages surrounding the PSU and in the epidermis of inflammatory acne lesions (19, 20). In addition, *C. acnes* induces IL-1β secretion and inflammasome activation *via* NLR family pyrin domain containing 3 (NLRP3) and caspase-1 in monocytes and sebocytes (21–23). The antimicrobial peptides, cathelicidin, and histone H4 can also be secreted locally in response to *C. acnes*. Histone H4 secreted by sebocytes exerts direct microbial killing, while cathelicidin interacts with components of the innate immune system within the acne microenvironment, such as psoriasin and beta-defensins, all of which contribute to the direct killing of *C. acnes* (24, 25).

## CD4^+^ T Cells in the Skin

In the periphery, naive CD4^+^ T cells undergo differentiation into distinct T cell lineages upon interaction with APCs and the influence of specific cytokines. These distinct lineages play a major role in mediating immune responses mainly through the secretion of specific cytokines and antimicrobial molecules. The CD4^+^ T cells perform multiple functions ranging from the activation of innate immune cells, B cells, cytotoxic T cells, as well as other non-immune cells. They also play a critical role in the quelling of immune reactions (26).

Traditionally, the CD4^+^ T cell subset lineages have been classified based on the cytokines they produce expression of characteristic transcriptional factors (TFs). Based on this classification, CD4^+^ T cells have been designated into T-helper-1 (T_H_1), T-helper 2 (T_H_2), T-helper 17 (T_H_17), follicular helper T cells (Tfh), induced T-regulatory cells (iTregs), regulatory type 1 cells (Tr1), and T-helper 9 cells (T_H_9) (27–33). In recent years, the diversity of T_H_ subsets has increased in complexity and the designation of T_H_ subsets beyond T_H_1, T_H_2 and T_H_17 cells remains a subject of intense debate as the traditional classification system fails to account for T_H_ cells that are involved in the induction of various pathologies. Of note, the emergence of novel technologies has enabled the simultaneous measurement of several cytokines at once along with other markers such as TFs, chemokine receptors and integrins at the single cell level, making it impossible to categorize T_H_ cells based on a dominant cytokine or even a family of cytokines (34). The new proposed paradigm reorganizes and expands the T_H_ universe based on the help these cells provide to the actual cell targets, rather than on the transient expression of certain cytokines and TFs (35). The debate on taxonomy to capture the complexity and diversity of T_H_ cells is described elsewhere (35–37).

Functionally, T_H_17 cells produce IL-17, IL-22 and IL-26 and play an important role in the clearance of extracellular bacteria and fungi from epithelial surfaces (38–40). T_H_17 cells are an important component of T cell immunity as patients with loss-of-function mutations in genes coding for IL-17, IL-17 receptor and/or the transcriptional factor RORγt have been shown to suffer from recurrent infections of the skin, nails, and mucosal surfaces. IL-22 can cooperate with IL-17 to activate epithelial cells to produce antimicrobial peptides (41–43). Finally, IL-26 is a cationic antimicrobial protein that kills extracellular bacteria by creating pores on the bacterial membrane (39). Like other T_H_17 cell cytokines, IL-26 is highly expressed in the skin lesions of psoriatic patients (44), colonic lesions in patients with inflammatory bowel disease (45, 46) and in the synovial fluid of individuals with rheumatoid arthritis (47, 48). A risk locus containing *IL26* and single-nucleotide polymorphisms within the *IL26* gene region have been associated with multiple sclerosis (49), highlighting the fact that IL-26 may be involved in driving T_H_17 cell–associated inflammatory activity.

IL-17 is a key cytokine involved in the recruitment, activation and migration of neutrophils to sites of inflammation (50, 51). Recruitment of neutrophils not only results in the phagocytosis and clearance of microbes, but can also result in tissue destruction. Similar to neutrophils, T_H_17 cells may also contribute to host defense and/or tissue damage as a result of uncontrolled TET activation and release. We envisage a scenario where an appropriate and timely decline of T_H_17 responses within the dermis may be required to minimize tissue injury or damage. In essence, this decline in T_H_17 response may be achieved by a shift toward production of cytokines such as IL-10 by T_H_17 cells. It is therefore likely that, in the steady-state, skin resident T_H_17 cells may have the capacity to adjust their cytokine profiles and secrete a combination of cytokines such as IL-26 and IL-10 that together can help dampen the inflammation and simultaneously time aid in combatting diverse skin pathogens, such as *Staphylococcus aureus* (*S. aureus*) and *C. acnes*, as needed. Thus together, with other innate immune cells, rapid cytokine secretion and TET formation may represent an efficient local host defense for controlling pathogenic microbes on the skin.

**Figure 1:**
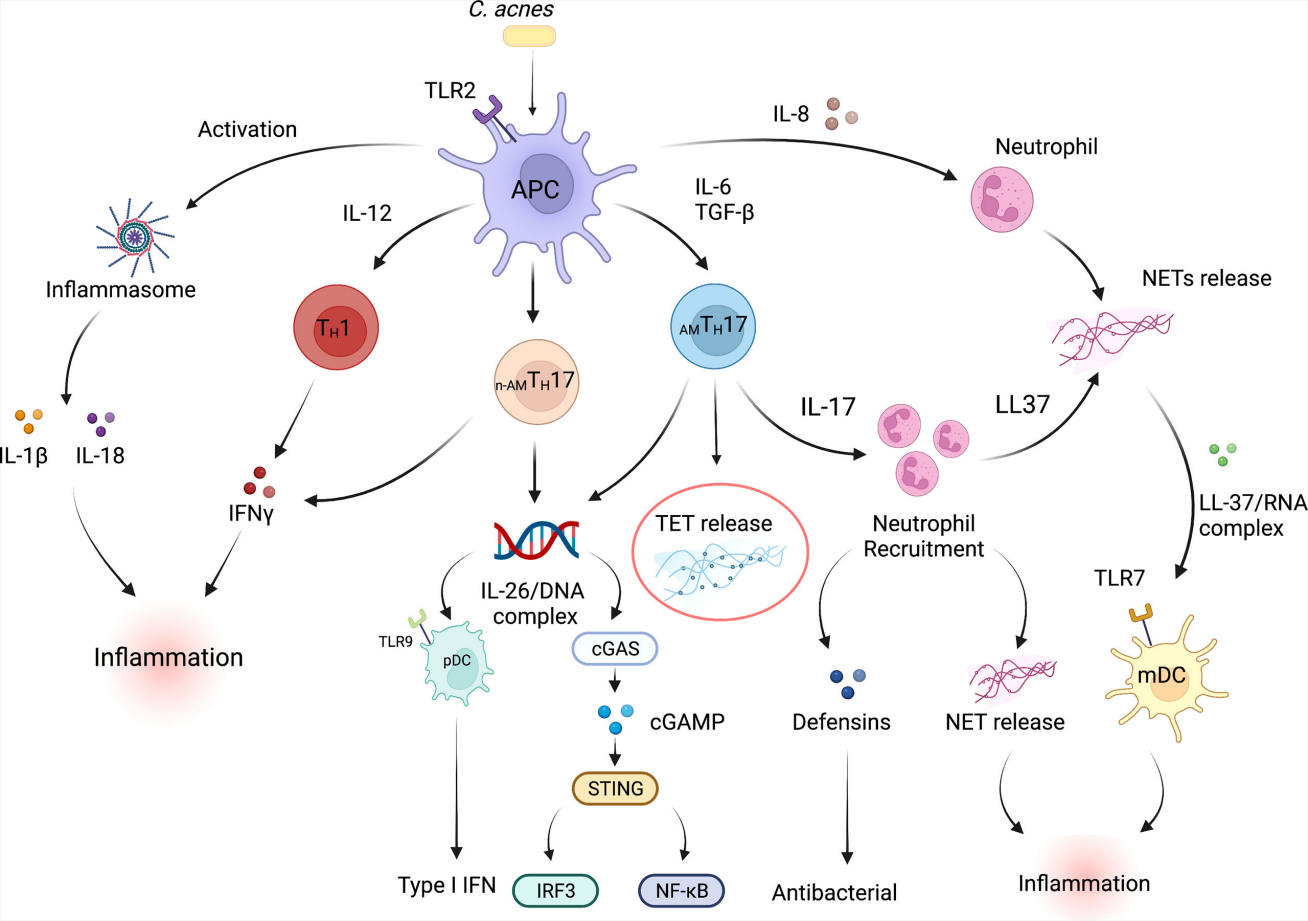
Inflammatory and antimicrobial pathways in acne vulgaris. Multiple interconnected innate and adaptive immune pathways contribute to skin inflammation associated with acne. *C. acnes* is the pre-dominant organism in the sebaceous region of the skin. Dysbiosis of the skin microbiome, *C. acnes* colonization or a disrupted homeostasis can upregulate the production of pro-inflammatory cytokines by skin-resident APCs such as dendritic cells, including high levels of IL-1β, IL-6, IL-8, IL-12p70, and TGFβ. Cytokines such as IL-6 and TGFβ induce naive T cells to differentiate into effector T_H_17 cells, whereas IL-12 drives a T_H_1 differentiation program. *C. acnes* strains within the pilosebaceous unit can also influence CD4^+^ T cell differentiation; *C. acnes* strains-associated with healthy skin promote the differentiation of naïve T cells into IL-10-producing _AM_T_H_17 cells whereas acne-associated strains promote the development of a non-antimicrobial T_H_17 subpopulation (_n-AM_T_H_17). IL-8, IL-17, IL-26, LL-37 and TET production by immune cells within acne lesion promote neutrophil recruitment and increased antimicrobial activity. Expression of defensins can also drive antibacterial action. On the other hand, NET/TET release and formation of LL-37/RNA and/or IL-26/DNA complexes can activate both myeloid and plasmacytoid DCs leading to the production of proinflammatory cytokines that further promote inflammation (39). mtDNA can also activate the cGAS-STING pathway and production of type I IFNs (58, 59). *C. acnes* can also activate TLR2 receptor expression on macrophages and subsequent secretion of IL-1β and IL-18 secretion in an inflammasome-mediated fashion. IFN-γ release by T_H_1 cells further drives the inflammatory responses within acne lesions. In addition, proinflammatory conditions exist when IFN-γ-producing T_H_1 cells are exposed to TNF-α and IL-12. APC, Antigen presenting cell; TGFβ, Transforming growth factor- Beta; pDC, plasmacytoid dendritic cell; IFN-γ, Interferon gamma; TLR, Toll-like receptor; mDC, myeloid dendritic cell; cGAS-STING, cyclic GMP-AMP synthase (cGAS)-stimulator of interferon genes (STING) pathway. Created with Biorender.com.

The components of both the innate and adaptive immune system have been demonstrated to play a part in the inflammatory responses that are observed during acne pathogenesis. Notably, in an elegant histological study of acne lesions, Norris *et al.* demonstrated that lymphocytes were the predominant cell types, with CD4^+^ T cells being among the cells that were detected within the early inflammatory infiltrate surrounding acne lesions (15). Polymorphonuclear neutrophils (PMNs), were increasingly evident at 24 and 72h and were linked to the disruption of the PSU. Follow up studies from our group and others further demonstrated that both T_H_1 and T_H_17 associated cytokines such as IFN-γ and IL-17 were prominent in acne lesions (4, 52, 53), and may contribute to either the inflammatory and/or to antimicrobial activity observed in acne. In early lesions, therefore, it is likely that antimicrobial activity may be driven by T cell-derived cytokines, that ultimately lead to bacterial lysis. The release of microbial components upon bacterial lysis may also lead to direct activation of the innate immune response, further enhancing inflammation. Moreover, T_H_17 cells recruit neutrophils, which in inflamed lesions, infiltrate around hair follicles and phagocytose *C. acnes* (54). Additionally, neutrophils can also release reactive oxygen species (ROS) and lysosomal enzymes that may further exacerbate acne disease (55–57). The proposed mechanisms by which immune cells interact with each other within an acne lesion are summarized in **Figure 1**.

This review focuses on the antimicrobial subpopulation of T_H_17 cells (_AM_T_H_17), whose antimicrobial capacity were discovered using *C. acnes* as a model organism. Moreover, our recent advances in T_H_17 cell research have reshaped the concept of immunomodulatory CD4^+^ T cells and have shown that direct antimicrobial activity by _AM_T_H_17 subpopulations requires ET formation, which may be critical for pathogen clearance within the cutaneous environment (60).

## T_H_17 Differentiation

T_H_17 cells express the transcription factor RAR-related orphan receptor-γt (RORγt), and are induced by IL-6, TGFβ and IL-1β. The differentiation of the T_H_17 lineage has also been shown to be distinct from that of T_H_1 and T_H_2 cells. T_H_17 cells are induced by signal transducer and activator of transcription 3 (STAT3) and RORγt working synergistically with one another (61). The transcription factor, forkhead box P3 (FOXP3), is the negative regulator of RORγt and T_H_17 programming. FOXP3 maintains tolerance by inducing Regulatory T cells (Tregs) differentiation *via* STAT6 and downregulating differentiation of T_H_17 cells (62). However, the T_H_17/Treg balance is shifted in favor of T_H_17 when proinflammatory cytokines, such as IL-1β, IL-6 and IL-21, are present. TGFβ is a critical cytokine required for T_H_17 differentiation. TGFβ not only induces the differentiation of T_H_17 cells in conjunction with IL-6 in mice and IL-1β in humans, but also independently orchestrates the differentiation of naïve CD4^+^ T cells into Tregs (63, 64). Exposure of naïve CD4^+^ helper T cells to TGFβ/IL-1β and IL-6 results in the inhibition of FOXP3 and subsequent RORγt activation, thus initiating T_H_17 differentiation and programming (63, 65–67). RORγt activation therefore promotes both the expression of IL-17 and IL-23 receptor (68). Interestingly, we have demonstrated that *C. acnes* stimulates the expression of both RORγt and RORα in peripheral blood mononuclear cells (PBMCs) suggesting that *C. acnes* contributes to T_H_17 differentiation (4, 69). In addition, we and others have demonstrated that IL-23, by itself, does not induce the development of T_H_17 cells from naïve CD4^+^ helper T cells, but instead IL-23 is important in the maintenance and survival of T_H_17 cells (70–72). IL-21, which belongs to the IL-2 family of cytokines, further amplifies T_H_17 cell differentiation and stabilizes the development of T_H_17 cells in cooperation with Transforming growth factor B (TGFB) in an IL-6-independent manner (61, 73–75).

As highlighted above, TGFβ is critical for T_H_17 differentiation, but it also influences T_H_17 biological functions. Different isoforms of TGF have also been shown to induce distinct phenotypic functions in T_H_17 cells. As an example, in the absence of IL-23, the combination of TGFβ1 and IL-6-induced T_H_17 cells did not cause experimental autoimmune encephalomyelitis (EAE), but TGFβ3-induced T_H_17 cells were highly pathogenic (76). T_H_17 cells, themselves, exhibit high phenotypic and functional plasticity, which suggests that these cells can transdifferentiate into other T cell subsets that are dependent on the inflammatory context (77–79).

In summary, T_H_17 cell differentiation and regulation is mediated by a tightly controlled complex of cytokine and transcription factors, all of which results in both pathologic and/or non-pathologic immune effector functions.

## Diversity of *C. acnes* Strains and Influence on T_H_17 Differentiation

Similar to other bacterial species, *C. acnes* possesses phenotypic and genotypic diversity (70), and the influence of this diversity at the strain level and its connection to either healthy skin or acne is unclear (80). As observed in other human diseases, recent typing of *C. acnes* strains revealed that not all strains are pathogenic. Certain *C. acnes* strains were associated with acne, while others were associated with healthy skin (80–84). Strains of *C. acnes* also show differences in their pathogenic potential and secretome profiles (85–87), as they differ in their ability to induce human β-defensins, influence cell growth, and activate both innate and adaptive components of the immune system (88–92).

We have demonstrated that *C. acnes* strains differentially modulate the fate of T_H_17 cells (70). Some healthy-skin associated *C. acnes* strains induce T_H_17 subpopulations that produce the anti-inflammatory cytokine IL-10, thus downregulating their pathogenic functions (70, 93). T_H_17 cells in the gut mucosa have also been shown to transdifferentiate to IL-10-producing regulatory T cells (Tr-1 cells), a process seemingly reliant on aryl hydrocarbon receptor (AhR) and TGFβ (40, 94). The ability of T_H_17 cells to secrete IL-10 has also been reported following treatment with TNFα inhibitors (95). IL-10 production by T_H_17 cells has been suggested to be under the regulation of several transcription factors, such as c-Maf and Aiolos (94, 95). In contrast, the majority of acne-associated *C. acnes* strains induce T_H_17 subpopulations with a pro-inflammatory phenotype that produce IL-17 and IFN-γ (70, 96).

## CD4^+^ T Cell–Mediated Killing of Target Cells

CD4^+^ T cell–mediated killing of target cells is well described in the literature, but the extent to which T_H_1 and T_H_17 CD4^+^ T subsets mediate antimicrobial activity is unknown (60, 97). In the case of the T_H_17 cells, their role in pathologic inflammation and disease is well-defined (73, 98), yet it is unclear what distinguishes the inflammatory T_H_17 cells elicited by pathogens from those within the PSU or the inner layers of the dermis that are induced by commensals. Two subpopulations of T_H_17 cells that are functionally different were demonstrated to simultaneously reside within the gut microenvironment during pathogen-induced inflammation. In particular, commensal filamentous bacteria within the gut were shown to induce T_H_17 cells that were non-inflammatory and homeostatic in nature. In contrast, *Citrobacter rodentium*, a model pathogen for gastrointestinal human disease research, induced T_H_17 cells that were highly inflammatory and feature a distinct cytokine profile that likely contributes to pathogenic intestinal pathologies (99). Similar to gut microbes, acne-associated and healthy-associated strains of *C*. *acnes* differentially regulate CD4^+^ T cell responses to induce T_H_17 cells that secrete either IL-17 and IFN-γ (pro-inflammatory) or IL-17 and IL-10 (anti-inflammatory) respectively (70). We and others revealed that *C. acnes* induce IL-17 and IFN-γ in CD4^+^ T cells, and that IL-17-secreting cells are visualized within the perifollicular infiltrates of acne lesions, which is consistent with the fact that T_H_17 cells may contribute to both the inflammatory and/or antimicrobial responses during acne progression (4, 52, 100).

More recently, while studying the antimicrobial mechanisms involved in T_H_17 cell–mediated killing of bacteria, we identified a subpopulation of T_H_17 cells that expressed and secreted multiple antimicrobial proteins. These findings were strengthened by the observation that this antimicrobial subpopulation, termed “_AM_T_H_17”, released histone-rich T cell extracellular traps (TETs) in conjunction with other antimicrobial proteins that entangled and killed *C*. *acnes* and other bacteria (60). This observation suggested that _AM_T_H_17-mediated killing of bacteria is likely to be a general feature that is essential for the homeostatic control of bacterial colonization. By contrast, a separate subpopulation of T_H_17 cells that were induced by acne-associated strains did not exhibit antibacterial activity (_n-AM_T_H_17) against Gram-positive and Gram-negative bacteria.

## T_H_17 and Extracellular Traps

Secretion of extracellular traps loaded with DNA is likely an ancient, conserved function of the innate immune system (101). The discovery and observation that neutrophil extracellular traps (NETs) trapped and killed bacteria changed our collective thinking of the role of these cells in host defense mechanisms, above and beyond the traditional function of microbe phagocytosis cytokine, secretion and antimicrobial peptide release (102). Now, we know that ETs are part of the arsenal of several immune cells including basophils, mast cells, eosinophils, macrophages, and T_H_17 cells (60, 103–112).

The first observations of lymphocytes extruding DNA were made in 1972. It was difficult to dissect the contribution of extruded DNA in immunity, and it took close to 50 years of intensive investigation for researchers to identify the lymphocyte populations that were capable of releasing ETs. Taking advantage of the fact that NETs are formed in patients with systemic lupus erythematosus (SLE), Rocha Arrieta et al. discovered that both B and T cells had the ability to secrete DNA into the extracellular microenvironment in response to SLE serum and other inflammatory stimulants. These extracellular DNA were not considered as ETs since this study did not evaluate the proteins associated with DNA (113). Focusing on T cells, work by Costanza and colleagues further showed that following activation, murine CD4^+^ T cells were able to extrude DNA fibers that they termed, “threads” (114). This release of DNA “threads” from CD4^+^ T cells was also shown to be dependent on mitochondrial ROS (114). However, the responsible T cell subset remained unknown. From the work of our lab, we demonstrated that T_H_17 cells extrude fibrous DNA threads coated with antimicrobial molecules that trap bacteria. We termed these threads TETs (60).

**Figure 2:**
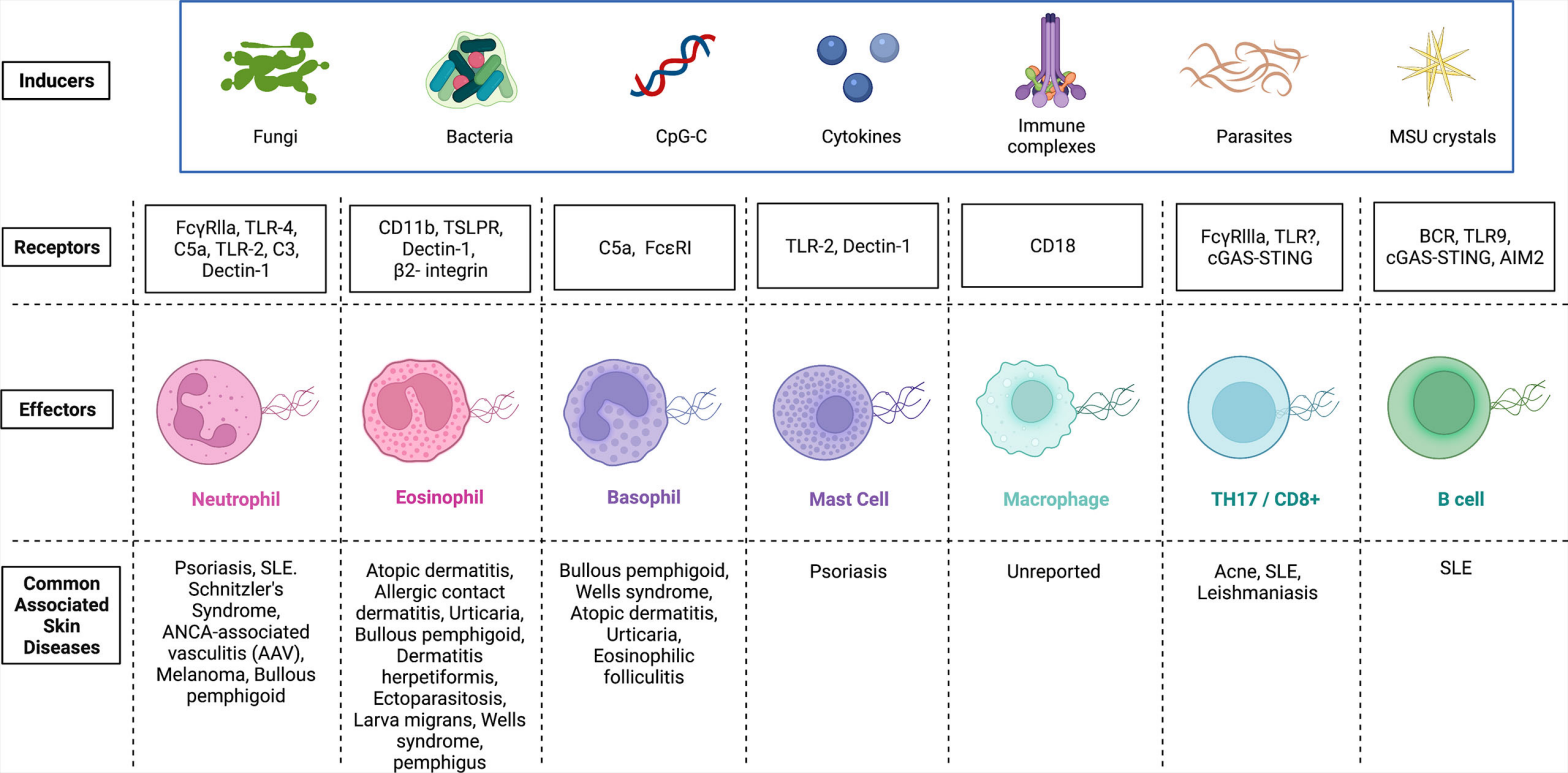
Inducers and potential receptors that activate the release of extracellular traps in immune cells and the associated skin diseases. Extracellular traps were first identified in neutrophils, and later, this phenomenon was detected in several other immune cells. Although there exists some overlap, inducers and receptors vary widely. Activation of immune cells by microbes, CpG-C, cytokines, immune complexes, parasites and MSU crystals can initiate the release of extracellular traps. Receptors on immune cells (Effectors) that have been identified and demonstrated to interact with inducers of extracellular trap formation are shown. Mechanisms by which ET formation are initiated in various effector cells vary and any dysregulation of each cascade can be pathological. Skin diseases associated with dysregulated extracellular trap formation are highlighted. FcγRs, Receptors for the constant region of IgG immunoglobulin; TLR, Toll-like receptor; C5a, Complement component 5a; C3, Complement component 3; TSLPR, Thymic stromal lymphopoietin receptor; FcϵRI, High affinity IgE; BCR, B cell receptor; STING, Stimulator of interferon genes; MSU, Monosodium urate; CpG-C, Synthetic short single-stranded DNA molecules made of cytosine triphosphate deoxynucleotide. Created with Biorender.com.

As previously reported, ETs are released by different immune cells and these ETs entrap not only Gram-positive/negative bacteria and group A streptococci, but also pathogenic fungi (60, 103–111, 115). Importantly, we observed that *C*. *acnes* can induce the _AM_T_H_17 cells, which upon activation are able to extrude TETs: fibrous DNA structures that are strikingly decorated with histones and antimicrobial molecules. TETs released by _AM_T_H_17 cells form interlaced structures in the extracellular space and entangle *C*. *acnes* (60). After entrapment, most of the *C*. *acnes* were killed (60). However, it is important to highlight that some bacteria species, such as *Streptococcus pneumoniae*, have developed strategies to evade capture and killing by repelling cationic antimicrobial peptides (CAMPs) found within ETs or by degrading the DNA backbone of ETs with enzymes such as, DNases (115–117). DNase treatment inactivates TETs and renders them ineffective, suggesting that DNA is required for TET structure and function. During acne pathogenesis, disruption of the PSU allows for the entry of *C*. *acnes* into the dermis, which initiates an inflammatory response. Using confocal microscopy, we visualized TETs *in vivo* in biopsy specimens from acne lesions, observing the colocalization of fibrous structures composed of DNA and histone H2B in proximity to CD4^+^ T cells secreting IL-17 (60). The potential receptors on T cells that mediate interactions with bacteria leading to TET induction are currently unknown (**Figure 2**).

## Histones and Extracellular Traps

Our findings were the first to demonstrate that _AM_T_H_17 cells make TETs loaded with histones H2B and H4 and that these _AM_T_H_17 cells secrete molecules, which could kill different bacterial strains (60). Through confocal imaging of acne lesions, we were able to detect TETs that were loaded with histones H2B, suggesting that ETs may play an important role in the clearance and elimination of pathogens within the extracellular matrix during acne disease progression (60). In unstimulated _AM_T_H_17 cells, we discovered that core histones, such as H2A, H2B, H3 and H4, had similar levels of expression. However, in TETs, H2B and H4 were found in higher concentrations than H2A and H1, and antibodies to histones H2B and H4 led to 60% reduction in TET-mediated bacterial killing (60). Similarly, neutralizing antibodies against core histones H2A and H2B interfered with NET-mediated bacterial killing and elimination of bacteria, further highlighting the importance of histones (102). Since ETs and histones in NETs and TETs can mediate injurious effects, a fine balance is needed between the host defense benefits and adverse effects that can be orchestrated by ETs (60, 118).

The antibacterial activity of histones is well documented, with first reports of antibacterial activity of histones and histone-like proteins originating in 1942 (119). Four core histones (H2A, H2B, H3, and H4) form an octamer, around which DNA enfold within nucleosomes (120). Apart from being part of the nucleosome structures, these core histones also play an important role in protecting the host epithelial surfaces from microbial invasion (120). Histones are predominant in the ETs of neutrophils, eosinophils, basophils, macrophages and other innate immune cells (102, 121–123). Pioneering studies by Hirsch et al. demonstrated the antimicrobial effect of arginine-rich histones against bacteria (124). Subsequent studies, including ours, have also shown that lysine-rich histones have bactericidal activity (60, 120). Lysine-rich histones, such as histones H2A and H2B, are highly expressed on the placental epithelial surface and have been suggested to be important in providing fetal protection against *in utero* microbial infection (125). Additionally, histones H2A and H2B are also effective in the neutralization of endotoxin, a major structural component of Gram-negative bacterial cell walls, and an important virulent factor during microbial colonization (24). From our work, H2B-loaded TETs released by the _AM_T_H_17 cells are antimicrobial against *C. acnes, E. coli, Pseudomonas aeruginosa*, and *S. aureus* (60). Furthermore, _AM_T_H_17 cells also released granulysin. Importantly, histone H2B gene expression is highly correlated with granulysin activity, which was consistent with the observed antimicrobial action. However, how this increased antimicrobial response is activated *in vivo* is unknown (120). While histone H3 exhibits antibacterial activity against both *E. coli* and *S. aureus*, less is known about its antibacterial actions on other bacteria (24). Histone H4 mediates antimicrobial activity through disruption of the cell membrane. Human sebocytes, the cells comprising sebaceous glands, can release H4, which is bactericidal against *C*. *acnes* (24). We observed the expression of the arginine-rich histone H4 in _AM_T_H_17 cells (60) and the antibacterial activity of H4 could be intensified by free fatty acids found on acne skin (24).

## Formation of Extracellular Traps (ETs) by Neutrophils

Immune cells are able to fight pathogens using various modes of action. They can secrete antibacterial proteins stored in granules, phagocytose microbes and then kill them intracellularly by antibacterial proteins or reactive oxygen species, or form and release ETs. The most well-characterized cells to secrete ETs are neutrophils (NETs). In addition to their phagocytic function, neutrophils utilize NETs for extracellular microbial killing (102). Different mechanisms are involved in NET formation. Generally, NET formation is a cell-destructive process, during which disassembly of nuclear material and disintegration of intracellular organelle membranes occur. After rupture of the plasma membrane, release of DNA, granular and cytoplasmic proteins (e.g. MPO and elastase) can facilitate extracellular killing of various pathogens, such as bacteria and fungi. Importantly, microbes and microbial components can induce NET formation (102, 116, 126–131). These inducers include bacteria or their structural components, such as lipoteichoic acid, lipopolysaccharides and bacterial breakdown products (132).

## Proposed Mechanisms of ET Release by B and T Lymphocytes

The first reported descriptions of lymphocyte-derived ETs demonstrated that both T and B cells secreted DNA following stimulation with SLE serum and other inflammatory stimuli (113, 133). The treatment with anti-IgM + lipopolysaccharides (LPS), which mimics stimulation of B cells under physiological conditions, initiated the release of DNA into the extracellular milieu, which suggests that ET release by B cells occurs *in vivo* (113). With regard to B cells, immune complexes can induce Fc*γ*RIIB activation independent of BCR-specific antigen binding (134, 135). Immune complexes can similarly mediate BCR cross-linking and induce downstream signaling pathways, promoting the secretion of ETs. DNA produced by ionomycin-stimulated peripheral blood B cells had high molecular weight, with no observed random or internucleosomal fragmentation that is observed in necrosis or apoptosis, respectively (136). Thus, B cell released DNA has similar characteristics to NETs (137, 138). Notably, the overall purity of B cell preparation was 80%, and therefore future work should examine the composition of the DNA extruded into the extracellular environment by B cells. The identification of proteins including histone composition or citrullination status will further help define the B cell-derived ETs (113).

ET formation in CD8^+^ T lymphocytes has been well characterized (139). Koh et al. demonstrated that upon activation of CD8^+^ T cells with α-CD3/CD28, these cells released ETs that they termed lymphocyte extracellular traps (LETs). LETs were shown to be morphologically different from CD4^+^ T cells-induced ETs as they appeared as long thin filaments that connected neighboring cells together. In contrast, CD4^+^ T cell-derived ETs formed a diffused patterns that appeared as a halo. Importantly, released LETs were loaded with CD107a^+^ cytotoxic vesicles that could kill distant target cells. The downside of this cytotoxic mechanism was that LETs release by CD8^+^CD107a^+^ T cells was associated with increased inflammatory infiltrates and subsequent severe disease in patients with cutaneous and mucosal leishmaniasis, suggesting that LETs could drive disease pathology within leishmania lesions. However, this study did not identify the CD8^+^ T cell subpopulations that were responsible for LET release (139).

Costanza et al. demonstrated that activated human and mouse CD4^+^ T cells produce extracellular DNA, which they termed, T helper-released extracellular DNAs or threads (114). However, the responsible T cell subset also remained unknown. Using RNA-seq and functional analysis of CD4^+^ T_H_17 cells, we demonstrated that _AM_T_H_17 cells, but not the _n-AM_T_H_17, T_H_1 or T_H_2 cells, released TETs that trap bacteria (60). After entrapment, most of the bacteria were killed (60). Further, T cell-derived DNA strands contained both mitochondrial and nuclear DNA, suggesting that both are involved in the process of TET formation (60, 114). Further exploration to determine whether the formation of TETs (TETosis) resembles vital (involving vesicular exportation or mitochondrial DNA) or a suicidal process is needed. However, it is plausible that several mechanisms contribute to the release of DNA traps by CD4^+^ T cells. These mechanisms are discussed below.

## Vital TETosis

**Figure 3:**
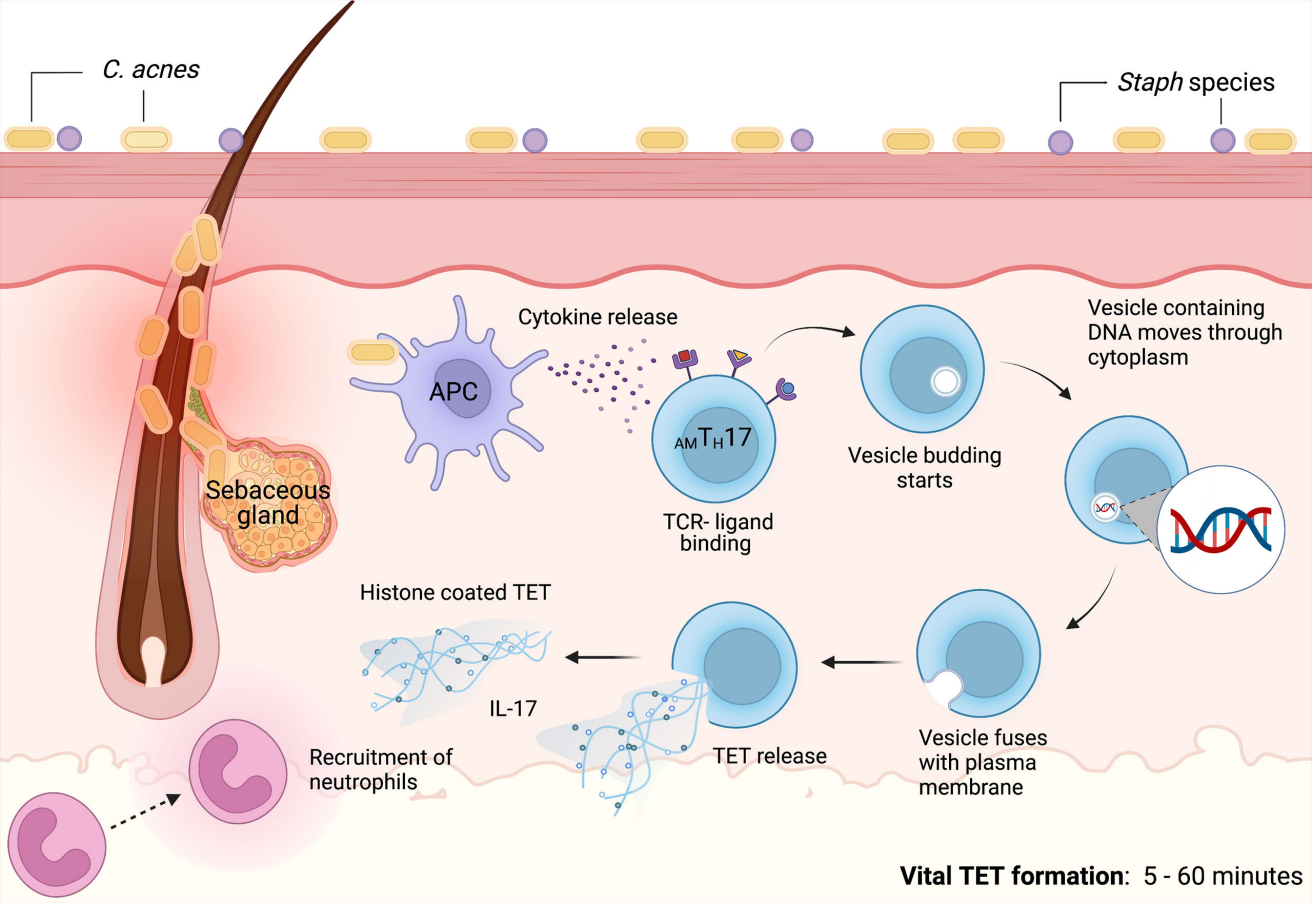
Proposed mechanism of vital TET formation in acne. In the pilosebaceous follicles, *C. acnes* strains trigger antigen presenting cells to secrete cytokines that induce naïve T cells to differentiate into distinct T cell subsets. Upon induction, _AM_T_H_17 cells can further be activated by bacterial ligands, PMA and other stimuli. TCR-ligand binding induce the formation of vesicles containing DNA. Blebbing of T cell nuclear envelope, vesicular exportation and exocytosis of vesicles containing DNA generate extracellular traps while preserving the integrity of the cell. As a result, _AM_T_H_17 cells retain conventional functions of viable T cells such as cytokine secretion. *In vitro*, we have observed that _AM_T_H_17 cells release TETs as early as 30 minutes after stimulation with *C. acnes*. Both the released TETs and IL-17 can recruit neutrophils to inflamed acne lesions. TETs trap *C. acnes* and are also loaded with antimicrobial molecules, including granulysin and histone H2B, H3 and H4 that kill bacteria. Created with BioRender.com.

Contrasting prior studies detailing the mechanisms of NETosis as a process that required several hours, we noted that *C. acnes*-stimulated TET formation in _AM_T_H_17 cells occurred within just 30 min (60). These TETs were likely induced by the recognition of bacteria or bacterial products. Using confocal microscopy, we demonstrated that activated _AM_T_H_17 formed TETs comprised of DNA decorated with histones, proteases and cytosolic proteins. These proteins ensnared bacteria, as well as provided large concentration of antimicrobial molecules, such as granulysin, that assist in trapping and killing of bacteria. This phenomenon has also been reported in neutrophils, as neutrophils that release NETs remained impermeable to SYTOX Green, suggesting that their structure remained intact. In neutrophils, vital NETosis has clearly been demonstrated by Pilsczek et al. as a process that involves vesicles of DNA budding off from the nuclear envelope, and delivered out of the cells without membrane perforation (140). Likewise, T_H_17 cells also remain structurally intact after TET formation, although additional studies are required to elucidate the precise mechanisms involved in vital TETosis. However, based on the similarities with NETs, we envisage a process where bacteria induce TET release through an initial blebbing event that is followed by substantive protrusions of the nuclear envelope. This is followed by transient detachment of the blebs/vesicles and transport of the vesicles through the cytoplasm and subsequent release of the DNA-loaded ETs *in vitro* and *in vivo* (133, 140–142). As a result, this proposed mechanism preserves the structural integrity of the T cell plasma membranes **(** **Figure 3** **).** It still remains unclear whether a T_H_17 cell that has ejected (parts of its) DNA is still viable and capable of performing T cell function, such as antigen recognition, immunomodulation, and cytokine secretion.

## Vital TET Formation: Mitochondrial DNA

**Figure 4:**
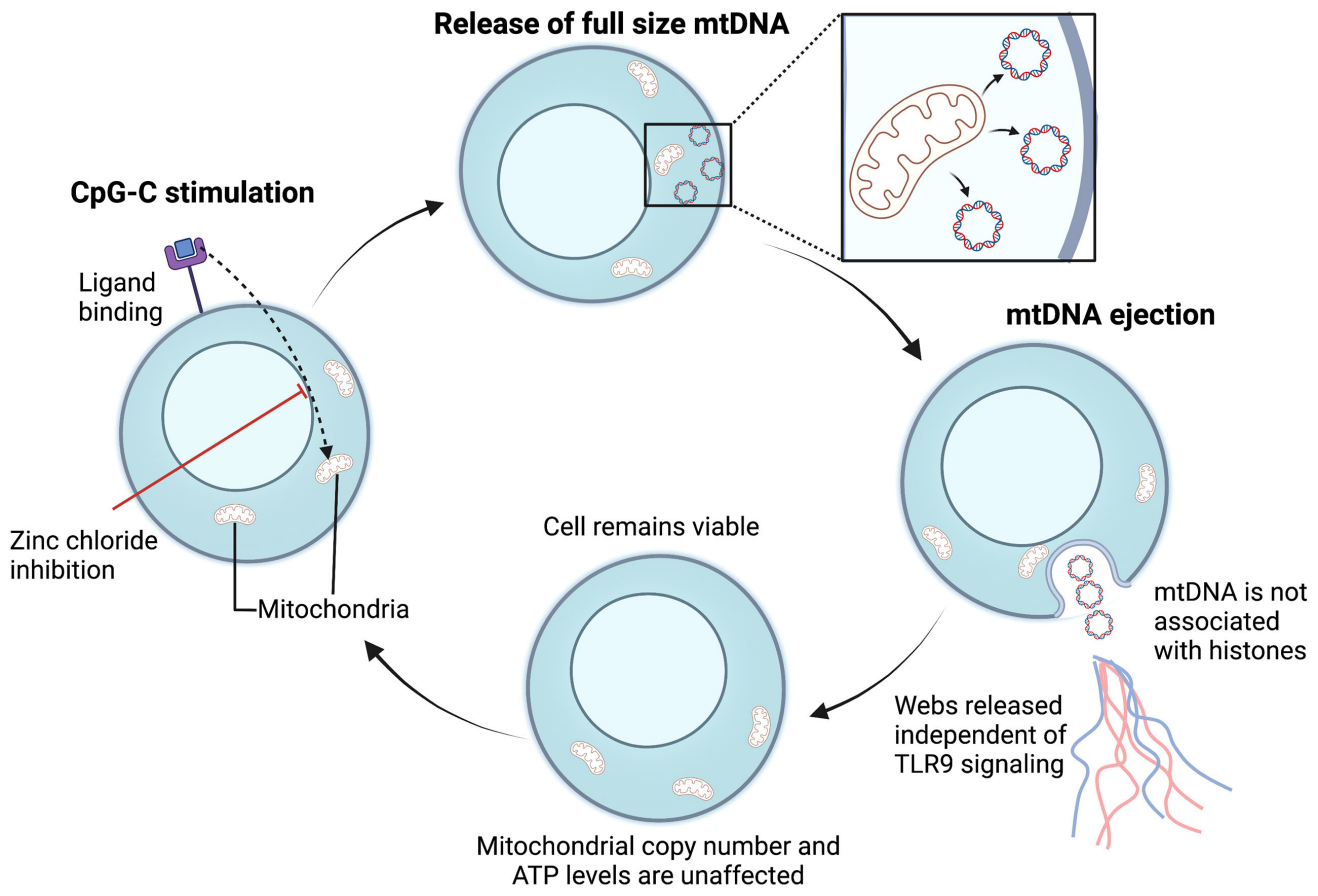
Proposed mechanism for mitochondrial vital TET formation. In B cells, CpG-C activation initiates the ejection of full-sized mtDNA that is free of histones (133). Free mtDNA enters the extracellular space in a web-like configuration, independent of TLR-9 activation. Following mtDNA ejection, the cell membrane is preserved, and the number of mitochondria remain unaffected. The cycle is repeated upon CpG-C re-stimulation and mtDNA release is an active process. Whether bacterial ligands can also induce ETs composed of mtDNA in T cells warrants further investigation. mtDNA, mitochondrial DNA; TLR, Toll-like receptor; ATP, Adenosine triphosphate; CpG-C, Synthetic short single-stranded DNA molecules made of cytosine triphosphate deoxynucleotide. Created with BioRender.com.

ETs produced in response to bacterial invasion have been shown to contain nuclear DNA as their critical structural component (143–146). However, other studies have reported ETs are composed of mitochondrial DNA (mtDNA) suggesting that the mitochondria can be involved in the process of vital ET formation and inflammatory response (147) (**Figure 4**). For example, GM-CSF primed neutrophils, upon stimulation *via* TLR4 or complement receptor 5a, produced ETs comprising of only mtDNA. In addition, *in vivo*, NETs containing mtDNA are seen in the serum of individuals post-trauma (148) and after orthopedic surgery (143). mtDNA-facilitated NET formation appears to be ROS-mediated (147). Interestingly, Yousefi et al. found ROS was essential for the release of mtDNA-NETs as mtDNA-NET formation was blocked when a ROS production inhibitor (diphenyleneiodonium) was used (147). However, the exact molecular mechanism of mtDNA-ET release is not fully understood in both neutrophils and lymphocytes. Whether mtDNA is involved in T_H_17-mediated TET formation remains to be determined.

mtDNA is a dominant driver of systemic inflammatory response after injury (143, 149, 150). It is recognized as an “alarmin” (151) that can stimulate the innate immune system at physiological concentrations. Free mtDNA from tissue and necrosis following injury or mtDNA-NETs could possibly re-activate neutrophils and other cells within the acne microenvironment through TLR2 and generate further ET production (19, 152). ET formation from neutrophils occurs through activation of TLR4 by another alarmin, more specifically, the high-mobility group box protein 1 (HMGB1) (153).

A recent study demonstrated a clear link between Severe acute respiratory syndrome associated coronavirus (SARS-CoV)-induced tissue damage in the skin and lungs of COVID-19 patients to the activation of the cyclic GMP-AMP synthase (cGAS- stimulator of interferon genes (STING) pathway and type I IFN signaling (58, 59). SARS-CoV-2 infection caused disruption of mitochondrial homeostasis in lung epithelial and vascular endothelial cells, leading to the accumulation of mtDNA. Both the mtDNA and DNA released from dying cells subsequently activated the cGAS-STING pathway and led to the secretion of type I IFNs and pro-inflammatory cytokines that further promoted hyperinflammation and tissue damage. This study clearly demonstrated that infections that disrupt mitochondrial function can lead to immunopathology. Whether the observed mtDNA was released by the process of vital ET formation is unknown. Additionally, the mechanisms and detrimental responses that might be induced by mtDNA and/or damaged DNA released from dying sebocytes and other cells within a disrupted PSU microenvironment in acne lesions are yet to be elucidated. Therefore, it is reasonable to speculate that mtDNA release by neutrophils, T_H_17 cells and other immune cells during chronic inflammation and/or after tissue injury could feed into a vicious cycle of immune activation. Such a vicious cycle could exacerbate the chronic inflammatory responses that are observed in acne (**Figure 1**).

## Suicidal TETosis

In neutrophils, suicidal NETosis is frequently initiated by ligand binding to neutrophil TLRs and receptors for IgG-Fc, complement or cytokines (102, 154, 155). Release of NETs was initially proposed to require the complete rupture of the neutrophil, a process that was termed NETosis (137). As such, NETosis is a cell death process, unlike apoptosis and necrosis, that relies on the generation of ROS by reduced nicotinamide adenine dinucleotide phosphate (NADPH) oxidase, *via* PKC and Raf-MEK-ERK signaling pathways (156–160).

Whether overstimulation of T cells with *C. acnes* can lead to suicidal/lytic TETosis in T cells within the acne microenvironment is currently unclear. Further investigations into the signaling pathways involved in TETosis are required. As has been observed in neutrophils, we speculate that the mechanisms by which TETs are formed will depend on the stimulus and the duration of the stimulation.

**Figure 5:**
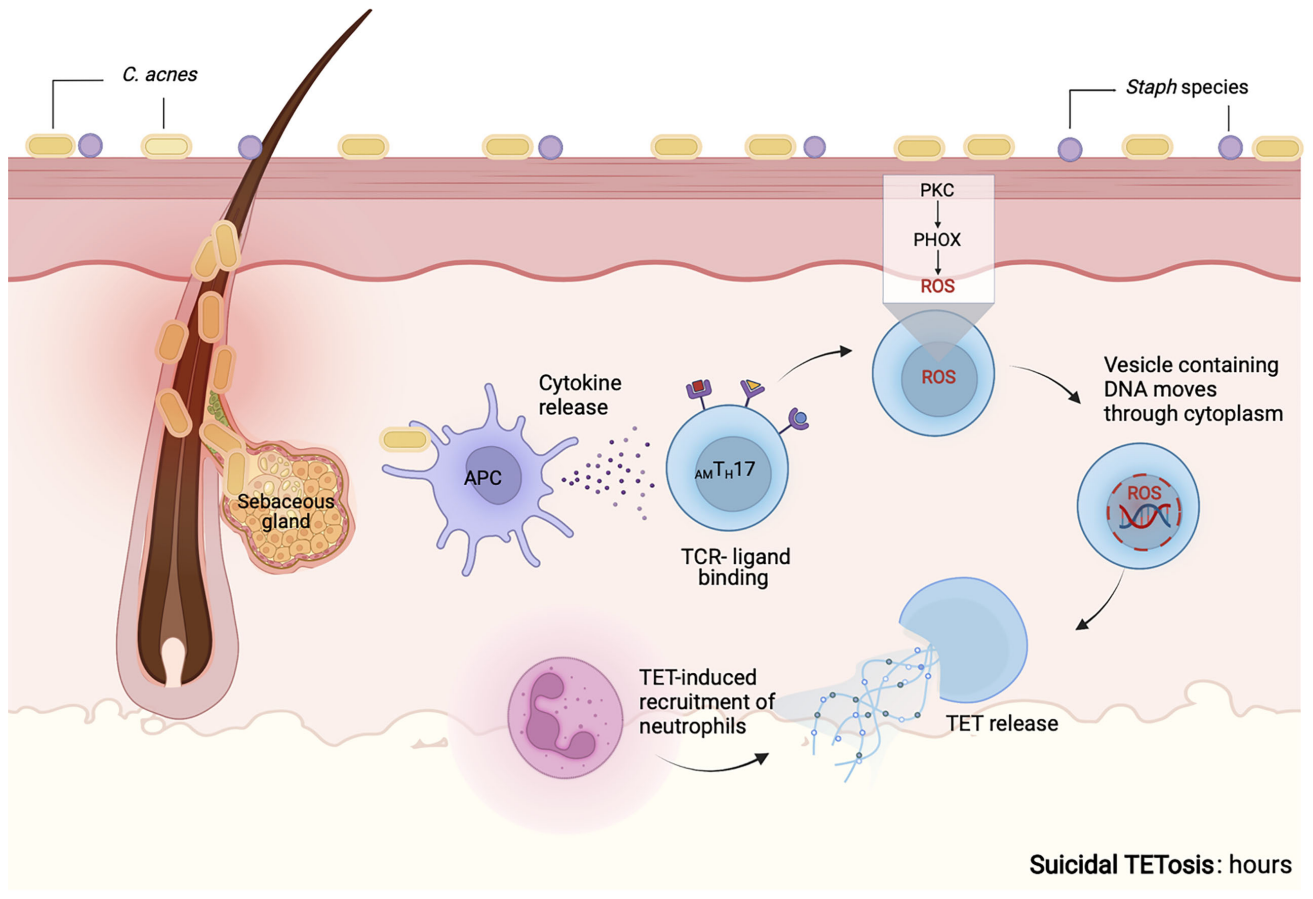
Proposed mechanism for suicidal TETosis. Similar to neutrophils, we propose that upon activation of TLR and receptors for IgG-Fc, complement or cytokine receptors on T cells, calcium is released from the endoplasmic reticulum into the cytoplasm. Higher levels of calcium within the cytoplasm, promote the activity of protein kinase C (PKC). This further induces the assembly of both the membrane-bound and cytosolic subunits of NADPH oxidase (phagocytic oxidase, PHOX) into functional complexes. This is followed by the release of reactive oxygen species (ROS), that causes the granules and the nuclear envelope to rupture. Following rupture both the nuclear and cytoplasmic contents are able to mix. Enzymes associated with the nucleus and the cytoplasm go on to degrade the linker histone H1, further processes the core histones (H2A, H2B, H3 and H4) and amplify the decondensation of chromatin. These events eventually lead to the rupture of the plasma membrane and the TETs are released into the extracellular space (160). The release of TETs can lead to T cell death and the loss of viable T cell functions such as antigen recognition and cytokine release. Enzymes that may mediate the proposed molecular processes are not known. Created with BioRender.com.

It has also been reported that S100A9-deficient neutrophils that are induced by *S. aureus* produce more mitochondrial superoxide, resulting in the release of NETs through suicidal NETosis. Increased suicidal NETosis failed to improve neutrophil removal of *S. aureus* in isolation. Rather, enhanced macrophage trapping and killing of bacteria was mediated by increased phagocytosis and the direct action of antimicrobial peptides (161). It has, therefore, been proposed that accelerated NET formation can be an immune-mediated mechanism that can amplify the antibacterial activity of macrophage (161). However, it is important to highlight that various pathogens such as *S. aureus* have developed survival mechanisms involving the secretion of nucleases and DNAses that allow them to evade and uncouple cooperation among immune cells through ET degradation (161). Our proposed mechanism of suicidal TETosis is illustrated in **Figure 5** and may likely resemble NETosis as previously described (156–160).

## The Duality of Extracellular Traps

Accumulation, incomplete removal, and improper localization of ETs can promote inflammation and cellular damage during infections and sterile inflammatory conditions (122). Although TETs are an important component of the host antimicrobial response against extracellular pathogens, we cannot preclude the possibility of TETs contributing to pathology. In psoriasis, NETs promote T_H_17 induction as part of the pathophysiology of the disease (162). Additionally, correlation of presence of NET-associated DNA with pathology has been implicated in other inflammatory skin diseases (**Figure 2**). Still, exploration of whether these observations are true, and whether *C*. *acnes*–induced TETs are part of the inflammatory cascade observed in acne is currently unclear (102, 163). In addition, it is plausible that the combined activity of CAMPs and histone-loaded TETs can lead to reduction of bacteria load within the PSU of acne patients. Further studies are needed to identify whether TETs are a double-edged sword in acne pathogenesis.

In summary, ETs have been implicated in diseases related to tissue injury: impaired wound healing, autoinflammation, autoimmunity, and tumor metastasis, highlighting their contributory role in numerous dermatological pathologies. Some select pathologies are discussed below.

## ETs in Tissue Injury and Wound Healing

Initially, it was proposed that ETs serve a protective function for the host by controlling infections. During pathogen invasion, these ETs can capture and degrade invasive microbes (164). However, additional studies have revealed that dysfunctional release and clearance of ETs can harm the host and contribute to disease (**Figure 2**). Structural components of ETs (i.e. decondensed DNA skeleton tangled with histones and granules) can function as damage associated molecular patterns (DAMPs), inciting additional tissue and organ injury (165–168). For example, the overabundance of histones decorating DNA filaments of traps promotes platelet aggregation and platelet dependent thrombin generation, resulting in disruption of circulation (159). Furthermore, NETs are associated with enzymes, myeloperoxidase (MPO) and elastase, that activate the immune system. When overexpressed, both enzymes can damage the epithelial barrier of the lung (169). Additional research is needed to elucidate whether TETs are associated with similar peroxidase and protease enzymes and whether these enzymes contribute to TET-induced tissue damage.

Degradation of the DNA backbone of ETs triggers the release of antibacterial proteins and upregulates inflammatory cytokines, such as type I interferon, IL-17, and IL-8 (170–173). While hyperinflammation is important for fighting infections, after injury, hypoinflammation is needed during tissue healing and repair (174). Therefore, overabundance of TETs during repair is counterproductive, unless there is continued need for protecting against inadvertent infections. In the case of acne, overabundance of TETs may be associated with hyperinflammation and tissue damage, contributing to acne scarring. It is plausible that skin conditions associated with aberrant wound healing, such as keloids, may be related to dysregulated TET formation and removal, but evidence on TET formation in keloids is currently lacking.

## ETs in Autoinflammation and Autoimmunity

Prior research demonstrates that NET components activate APCs, which present self-antigens to corresponding lymphocytes, leading to autoreactivity. Presence of ETs within the extracellular environment has been observed in various autoimmune diseases, including psoriasis, rheumatoid arthritis, and systemic lupus erythematosus (SLE) (175–177). In psoriasis, NETs, their citrullinated histones and antigens stimulate immunocompetent cells to produce antibodies against cytoplasmic antigens (ANCAs) and antibodies against nuclear antigens (ANAs) (175, 176). These antibodies act against neutrophil-associated enzymes and activate neutrophils to release more NETs (178). Autoantigens released by NETs become the target of successive antibodies perpetuating the development of autoimmune disease. In addition, abnormal NET formation and inefficient degradation can also initiate and/or facilitate autoimmune diseases (144, 169, 175, 176, 179). In SLE, ANCAs and ANAs lead to the generation of immune complexes and chronic activation of plasmacytoid dendritic cells (169). The inappropriate release of intracellular autoantigens during apoptotic cell removal caused continual production of autoantibodies; this cycle is considered the key pathogenic mechanism in SLE (154, 169). By binding DNA and cathelicidin (LL-37) of NETs, autoreactive antibodies form autoantibody-DNA/LL-37 complexes, which can be detected by other immune cells as a signal to “sustain” the autoimmune response (154). A relationship between inadequate NET degradation and SLE development has also been proposed (169). The ability to degrade NETs increased during SLE remission but decreased during relapse (176). Moreover, patients with increased SLE severity had higher NET antibody levels. In SLE, non-degraded NETs activate components of the complement system, such as C1q, which breaks down NETs. The opsonization of NETs and/or inhibition of DNase I activity were hypothesized to be responsible for this process (176). Thus, the continued presence and mis-regulated degradation of NETs contribute to the autoimmunity.

## ETs in Cancer

Identification of ETs in blood and solid tumors of animals and humans suggests that traps play a role in cancer. However, there exists conflicting evidence as to whether ETs serve as pro- or anti-tumoral factors (180, 181). For example, studies on NETs have shown that several NET components, such as MPO, proteinases, and histones are cytotoxic, whereas other studies have proposed that NETs promote metastasis (182–184). By increasing the concentration of LPS and upregulating the complement component, C3a, NETs induce more coagulation and tumorigenesis, further feeding into a positive-feedback loop that favors a pro-tumorigenic phenotype (185–187). These potential oncological mechanisms have been described for neutrophils but have yet to be studied in T cells. Given the crucial role of T cells in cancer, exploration of TETs in cancer is an exciting topic of interest.

Murine models demonstrate that ETs contribute to skin cancer and inflammation-mediated skin tumor cell growth (188, 189). NETs contribute to adverse reactions in murine melanoma models, regardless of whether they are spontaneous or immunotherapy-induced (190). In melanoma studies, IL-8 and type I IFNs act as potential therapeutic targets associated with NETs (191–193).

TETs have not been investigated in cutaneous cancers, such as cutaneous T-cell lymphomas (CTCL). For example, there is no evidence regarding ET production in Sézary syndrome (SS), an aggressive and rare leukemic form of CTCL (194). The chemokine CXCL8 is highly expressed in CTCL skin lesions and can act in concert with IL-8 and IL-17 to facilitate the priming and recruitment of neutrophils to the CTCL microenvironment. However, in CTCL patients, there is no evidence demonstrating the release of NETs within the CTCL tumor microenvironment, even though neutrophils isolated from the peripheral blood of these patients are phenotypically active (195). As the disease progresses, other cells such as myeloid-derived suppressor cells become activated and secrete ROS (196, 197). Increased ROS release has been linked to T cell tolerance and unresponsiveness within CTCL skin lesions (198). Additionally, if present within CTCL, neutrophils show compromised functionality, with diminished ingestion and intracellular killing of pathogens, as well as reduced NET production. Moreover, impairment of the host immune response against pathogens increases susceptibility to severe infections, and complications observed in CTCL patients (199, 200).

As the CTCL progresses, the tumor microenvironment is associated with increasing expression of T_H_2 TFs and cytokines (e.g. GATA-3, IL-4, IL-5, and IL-13) and declining levels of T_H_1 and T_H_17 associated TFs and cytokines (e.g. T-bet, RORγt, IL-12, IL-17 and IFN-γ) (201–207). Accordingly, late-stage CTCL is dominated by a T_H_2 tumor microenvironment and small numbers of T_H_1 cells and CD8^+^ T cells (205, 208–212). The increased T_H_2-bias is believed to be a key process that suppresses cellular immunity and anti-tumor responses in CTCL (213, 214). It has also been observed that different cellular sources of IL-17 may exist within the CTCL microenvironment. However, these IL-17-secreting cells fail to express the characteristic T_H_17 phenotype, indicating that IL-17 production may originate from dysregulated signaling and cannot be classified as a true T_H_17 response in SS (201, 215). Understanding how the low numbers of neutrophils, T_H_1 and T_H_17 TETs promote or inhibit tumorigenesis in CTCL may inform future development of skin cancer therapies and treatments.

## Research Gaps in TETs

Since the discovery of ETs two decades ago, substantial advances in defining their role in immune-mediated antimicrobial activity have been made (216). Processes related to their formation and mechanism of action are being identified. It is now generally accepted that ET formation is not unique to neutrophils as this phenomenon has also been observed in other immune cells (60, 103–112). Furthermore, work from our lab and others demonstrate that adaptive immune cells, namely antigen-specific T cells, extrude ETs suggesting that lymphocyte ETs serve as an important link between cells of the innate and adaptive immune system. Recent findings provide new perspectives on how T cell-derived ETs are involved in antimicrobial responses and influence host immune homeostasis. Though significant progress has been made regarding ETs, the complete role of TETs in host defense and inflammation is still unclear. More work is needed to elucidate the unique role of TETs and how that may inform treatment of ET-associated diseases.

Even though *in vitro* and *in vivo* formation of TETs has been observed, huge research gaps exist in our knowledge about how TETs form in general. Various particles and microbial agents can induce the release of histone-coated DNA ETs by T cells, but additional research is needed to identify the exact stimuli and receptors that induce TET formation (60, 113, 133, 139). Defining the distinctive characteristics of these stimuli is critical to advancing our understanding of diseases with TET-related pathophysiology. Equally important, research is needed to understand how ET formation is blocked. For example, the bacteria, *Lactobacillus rhamnosus*, inhibits NET release suggesting that different microbial species can act as agonists or antagonists of TET formation (217, 218). This brings up the interesting possibility that agents that modulate the microbiome, probiotics or antibiotics, may be beneficial in the management of TET-related diseases. An improved understanding of the role genetics play in TET formation and activity can also guide future therapeutics. Some NET associated diseases, such as psoriasis, have genetic component, so research efforts should pursue putative gene variants that predispose individuals to TET dysfunction. Understanding how host genetics influence TET activity may help us prognosticate individuals who are at risk for pathological conditions linked to extracellular traps.

Dissecting the mechanisms involved in TET induction and release may inform how TET activity can be moderated. With the introduction of novel computational and imaging algorithms that utilize high content screening-cellomics platform, efficient and precise detection of ETs are now possible (219). This high-throughput screening method uses cell membrane specific DNA dyes (permeable and impermeable) *in situ* to discern the cellular and morphologic characteristics of ET-forming cells. The combination of high-throughput screening with single-cell analysis of morphological changes within the nucleus and chromatin dynamics can provide precise detection of ET-forming cells with attention to specificity while eliminating user bias that is seen with other cell death assays. Furthermore, combining live cell imaging with staining for cell death markers *in situ* will help identify the specific death pathway and thus, discriminate between NETs, TETs, apoptosis, and necrosis (219).

An improved understanding of the uni-directional and/or bi-directional communication pathways between TETs and immune cells is also needed to fully understand the ultimate cellular response to TETs. We know that NETs do not always work in isolation. NETs can work cooperatively with macrophages in granuloma formation. NETosis by itself does not improve the killing potential of neutrophils against *S. aureus* but rather it augments macrophage microbial killing (161). Even though T_H_17 cells are adaptive immune cells,, the extracellular trap timing and response are more characteristic of an innate immune response; thus, T_H_17 cells may link the two arms of immunity by working cooperatively with other cells. T_H_17 cells recruit neutrophils and other proinflammatory cells to the infection site, where neutrophils activate and promote differentiation of T_H_17 cells, suggesting cross-talk between innate and adaptive cells (220, 221). These cooperative mechanisms have strong clinical relevance, as NETs have been connected to tumor progression and metastasis (185). Therefore, more research is needed to understand how T_H_17 cells coordinate host defense mechanisms with other cells.

Insights into how ET formation acts as a conduit for various immune cells, including neutrophils, T cells and macrophages, to fight microbes cooperatively within the deeper layers of the skin during acne disease is of particular importance. We have shown that _AM_T_H_17 cells release TETs that are coated with histones as part of the host antimicrobial defense. The fact that _AM_T_H_17 cells release ETs implies that this T_H_17 subpopulation can act as an important link between the innate and adaptive arms of the immune response, which ensures the efficient capture and destruction of invading microbes. However, there is a need to delineate the specific CD4 ^+^ T cell populations that have a capacity to release ETs, as the molecular markers that can be used to identify, purify and isolate TET-forming T cells are yet to be discovered. In the interim, it is possible that TET-forming T_H_17 have a specific pro-inflammatory signature as T_H_17 cells differentiated in the presence of IL-23 provoke EAE, but not T_H_17 induced by IL-6 and TGFβ alone (76). We are still in the beginning of understanding how the cytokine milieu influence the plasticity and heterogeneity of T_H_17 cells into either pathologic or non-pathologic conditions within different inflammatory settings. Yet, the verification of T_H_17 cell plasticity in humans and its functional importance implies that during pathological conditions, both anti-inflammatory and antimicrobial therapeutics should be designed to specifically target recruited cells while sparing T_H_17 subpopulations and other tissue-resident cells that promote skin tissue repair. Teasing apart, the likely, yin and yang activities associated with TET-forming T_H_17 cells will be of future interest (60).

Many unanswered questions remain when it comes to TETs. Future studies should focus on clarifying which pathologies, including autoimmune diseases, cancer, and chronic inflammation, that are associated with T cell derived ETs. In doing so, we may also begin to identify autoantigens that may be associated with TETs, thereby improving our understanding of the underlying mechanisms contributing to these diseases. Simultaneously, we will gain a greater understanding and appreciation for the critical protective role that TETs serve. As ET and TET research unfolds, therapeutic opportunities to exploit and control the formation, modulation, and regulation of these ETs for the treatment of inflammatory and autoimmune diseases may be on the horizon.

## Author Contributions

KO: data curation, formal analyses, data interpretation and manuscript writing; NO: data curation, formal analyses, data interpretation and manuscript writing; AN: manuscript writing/editing; GA: conceptualization, data curation, data analyses and interpretation, funding acquisition, methodology, project administration, resources, supervision, visualization, and manuscript writing/editing and submission. All authors edited the manuscript, gave final approval for publication, and agreed to be accountable for the work. KO and NO contributed equally to this work.

## Funding

This work was supported by NIH K01AR071479 (GA) and NIH R01AR081337 (GA).

## Conflict of Interest

The authors declare that the research was conducted in the absence of any commercial or financial relationships that could be construed as a potential conflict of interest.

## Publisher’s Note

All claims expressed in this article are solely those of the authors and do not necessarily represent those of their affiliated organizations, or those of the publisher, the editors and the reviewers. Any product that may be evaluated in this article, or claim that may be made by its manufacturer, is not guaranteed or endorsed by the publisher.
